# Experimental study of a new technique for minimally invasive percutaneous nephrolithotomy: intelligent pressure-controlled minimally invasive percutaneous nephrolithotomy

**DOI:** 10.1186/s12893-024-02361-y

**Published:** 2024-02-22

**Authors:** Zhongsheng Yang, Leming Song, Yongming Huang, Hua Chen, Ting Sun

**Affiliations:** 1 Department of Urology, The Affiliated Ganzhou Hospital, Jiangxi Medical College, Nanchang University, No. 16, Meiguan Road, Zhanggong District, Ganzhou City, Jiangxi Province 341000 China; 2 Department of Urology, The First Affiliated Hospital, Jiangxi Medical College, Nanchang University, No. 17, Yongwaizheng Street, Donghu Street, Nanchang City, Jiangxi Province 330006 China

**Keywords:** Renal pelvic pressure, Intelligent control, Suction, Percutaneous nephrolithotomy

## Abstract

**Background:**

To test the reliability and safety of a newly invented technique for minimally invasive percutaneous nephrolithotomy, intelligent pressure-controlled minimally invasive percutaneous nephrolithotomy (IPC-MPCNL).

**Methods:**

Eighteen kidneys of nine female pigs were randomly divided into three groups. Those in Groups A and B underwent IPC-MPCNL through the new system composed of a pressure-measuring MPCNL suctioning sheath and an irrigation and suctioning platform with pressure feedback control. The infusion flow rate was 500 ml/min in Group A and 750 ml/min in Group B. Those in Group C underwent MPCNL at an infusion flow rate of 500 ml/min. The renal pelvic pressure (RPP) monitored by a ureteral catheter and that monitored by the pressure-measuring sheath in Groups A and B were compared. The RPP in Group C was monitored by a ureteral catheter.

**Results:**

The RPP measured by the pressure-measuring sheath and that measured by the ureteral catheter in Group A was − 5.59 ± 1.95 mmHg and 4.46 ± 2.08 mmHg, respectively. The RPP measured by the pressure-measuring sheath and that measured by the ureteral catheter in Group B was − 4.00 ± 2.01 mmHg and 5.92 ± 2.05 mmHg, respectively. Hence, the RPPs measured by the pressure-measuring sheath in Groups A and B were consistent with those measured by the ureteral catheter. The RPP in Group C was 27.75 ± 5.98 mmHg (large fluctuations).

**Conclusions:**

IPC-MPCNL can be used to accurately monitor the RPP and maintain it within a preset safe range via suction. The new technique and the new system are safe and reliable.

## Background

Percutaneous nephrolithotomy (PCNL) has been used for more than 40 years to treat upper urinary tract stones, and it is still the method of choice for treating complex upper urinary tract stones [1]. As technology and equipment have advanced, various PCNL surgical techniques have been developed, and PCNL is evolving in the direction of precision and miniaturization [2, 3].

Common PCNL techniques include standard PCNL (24 to 30 Fr tracts), minimally invasive PCNL (MPCNL) (14 to 20 Fr tracts), Ultra-mini PCNL (UMP) (11 to 13 Fr tracts), and Super-mini PCNL (SMP) (10 to 14 Fr tracts). The UMP, SMP, and other MPCNL techniques are suitable for only certain types of upper urinary tract stones [4, 5]; therefore, they are not widely applicable. At present, the most widely used PCNL techniques are standard PCNL (24 to 30 Fr) and MPCNL (14 to 20 Fr) [6].

Although these two surgical techniques have their own advantages, they still have some problems [7–9]. For example, a low intraoperative infusion flow rate tends to cause a blurred surgical field, resulting in secondary injury, holmium laser-induced thermal injury, and low stone removal efficiency, whereas a high intraoperative infusion flow rate is likely to produce a high renal pelvic pressure (RPP) and serious related complications. New techniques need to be developed to improve the efficiency of stone fragmentation and removal while minimizing trauma.

**Fig. 1:**
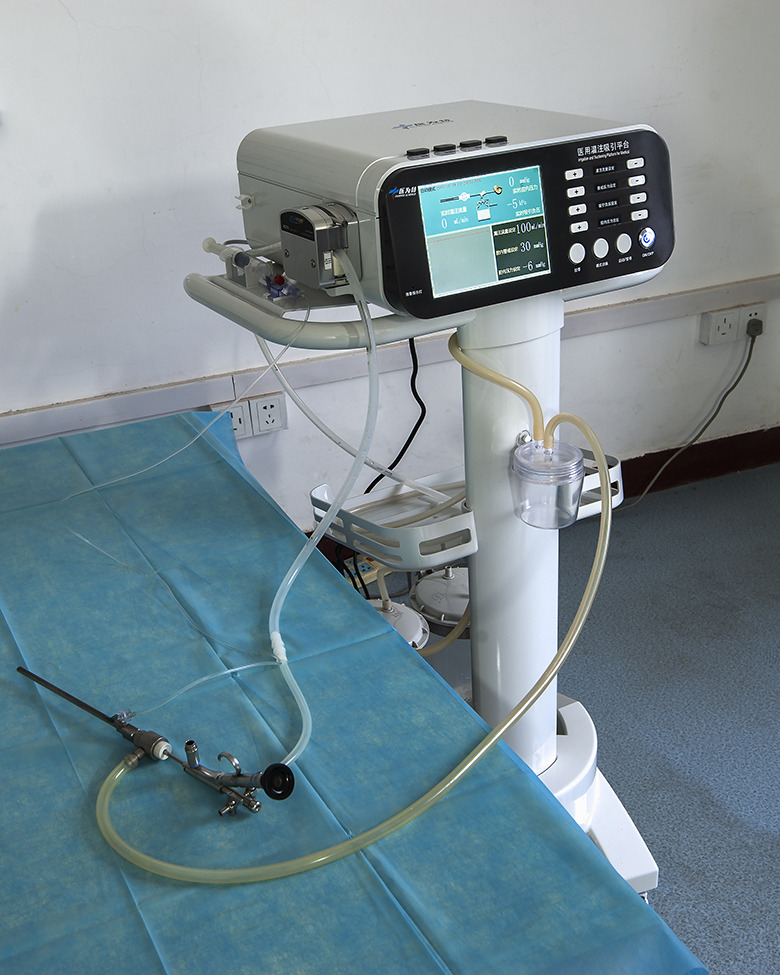
System developed for intelligent pressure-controlled minimally invasive percutaneous nephrolithotomy

To this end, after years of research and experiments, we have improved the PCNL device and developed a system that can automatically monitor and maintain the RPP within a preset safe range under different infusion flow rates. The system is composed of a pressure-measuring MPCNL suctioning sheath and an irrigation and suctioning platform with pressure feedback control (medical irrigation and suctioning platform). Using the new system, we designed a new surgical PCNL technique, i.e., intelligent pressure-controlled MPCNL (IPC-MPCNL), where IPC refers to intelligent control and monitoring of the RPP (Fig. 1). This technique uses less invasive minitracts during surgery and keeps the RPP within a safe range through suction and pressure monitoring and feedback, even at high infusion flow rates, making PCNL safer and more efficient. We performed IPC-MPCNL in living pigs, in which we monitored and recorded changes in the RPP under different infusion flow rates to test the reliability and safety of this technique in practical application.

## Methods

The experimental animals were nine female pigs weighing approximately 50 kg. The 18 kidneys of these pigs were randomly divided into three groups: two experimental groups (Groups A and B) and a control group (Group C). Those in Groups A and B underwent IPC-MPCNL using the new system at infusion flow rates of 500 ml/min and 750 ml/min, respectively, while those in Group C underwent MPCNL at 500 ml/min.

Experimental method: The experimental pigs were fasted for 6 h before the operation. After each pig entered the operating room, 0.2–0.3 mg/kg midazolam was injected into the gluteus maximus muscle. After 5–10 min, intravenous access was established via the marginal ear vein. After anesthetization with 2–3 mg/kg propofol, the pig was placed on an operating bed, and a ProSeal double-tube laryngeal mask was placed to preserve spontaneous breathing. A blood pressure cuff, electrocardiogram (ECG) electrodes, and an oxygen saturation probe were attached to the pig to monitor its vital signs. During the operation, propofol was injected at 6–10 mg/kg/h, and sufentanil was given intermittently at 2–3 µg/kg to maintain anesthesia.

In Groups A and B, the pigs were placed in the lithotomy position after successful anesthesia, and ureteral catheters were placed into the renal pelvis through bilateral urethras with a ureteroscope and fixed. The pigs were subsequently placed in the lateral position. Under the guidance of color Doppler ultrasound, an 18-G biopsy needle was used to sample renal tissue for histopathological examination, and a percutaneous renal tract was established by puncture. A pressure-measuring MPCNL suctioning sheath was placed into the tract. The pressure-measuring end of the sheath was connected to the input socket of the medical irrigation and suctioning platform through a disposable pressure sensor, and the suctioning end of the sheath was connected to the stone collection bottle of the medical irrigation and suctioning platform through a suction tube. A peristaltic tube was installed into a peristaltic pump, and the nephroscopic infusion tract was connected to the infusion tube of the medical irrigation and suctioning platform.

**Fig. 2:**
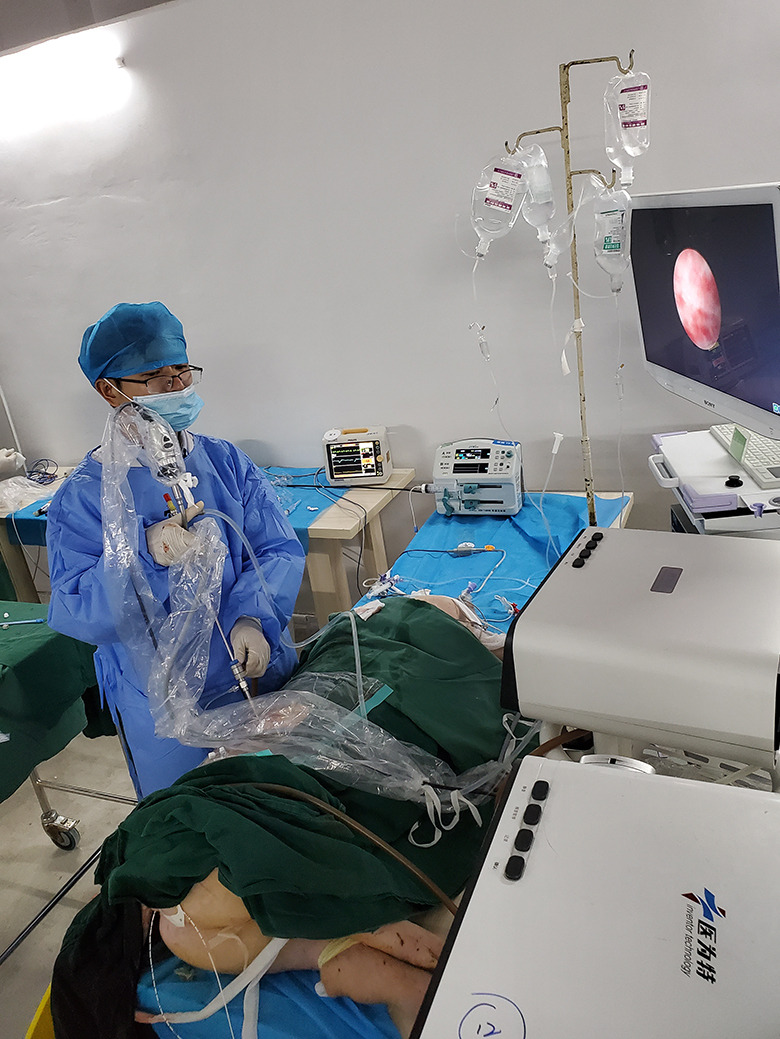
Intelligent pressure-controlled percutaneous nephrolithotomy

In the standby state of the control panel of the medical irrigation and suctioning platform, the mode was switched to automatic irrigation and suctioning mode, and the needed intraoperative infusion flow rate, intraluminal pressure control value, and intraluminal pressure warning value were set on the control panel of the platform. After the platform parameters were set, normal saline was injected via a 20-ml syringe to empty the air from the sensor and the pressure-measuring tube of the MPCNL suctioning sheath. After the intraluminal pressure was relatively stable, the “Zero” button was pressed to calibrate the intraluminal pressure to 0 (a fluctuation range of the intraluminal pressure within 2 mmHg indicates successful zero-calibration). After successful zero-calibration, the pressure in the kidney at the end opening to the atmosphere was set to 0 mmHg, and the actual pressure in the kidney was X cmH_2_O (i.e., 0.735X mmHg), due to the height difference (X cm) between the pressure-measuring end of the sheath and the kidney. In addition, the ureteral catheter of the ipsilateral kidney was connected to an ECG monitor through a pressure sensor for ambulatory blood pressure monitoring and recording. The pressure measured by the ureteral catheter was theoretically the sum of the pressure measured by the pressure-measuring sheath and 0.735X mmHg. After preoperative preparation, the medical irrigation and suctioning platform was turned on to perform IPC-MPCNL, and normal saline was infused for 60 min (Fig. 2).

In Group C, the pigs were placed in the lithotomy position after successful anesthesia, and ureteral catheters were inserted into the renal pelvis through bilateral urethras with a ureteroscope and fixed. The pigs were placed in the lateral position. Under the guidance of color Doppler ultrasound, an 18-G biopsy needle was used to sample renal tissue for histopathological examination, and a percutaneous renal tract was established by puncture. A 16-Fr peel-away sheath was placed into the tract, and a nephroscope was connected to an infusion pump to infuse normal saline.

Animals in each group were infused according to the preset infusion flow rate for 60 min, after which the RPP was recorded. After the operation, color Doppler ultrasound was performed to examine the kidneys and perirenal regions, and the kidney tissues were sampled again for histopathological examination.

## Results

During each operation, the ureteral catheters were successfully placed, and the percutaneous renal tract was successfully established. After the MPCNL sheath was placed in the tract to perform MPCNL, no major bleeding or other conditions affecting the operation occurred. The simulated operation and intraoperative RPP monitoring were completed in all pigs.

**Fig. 3:**
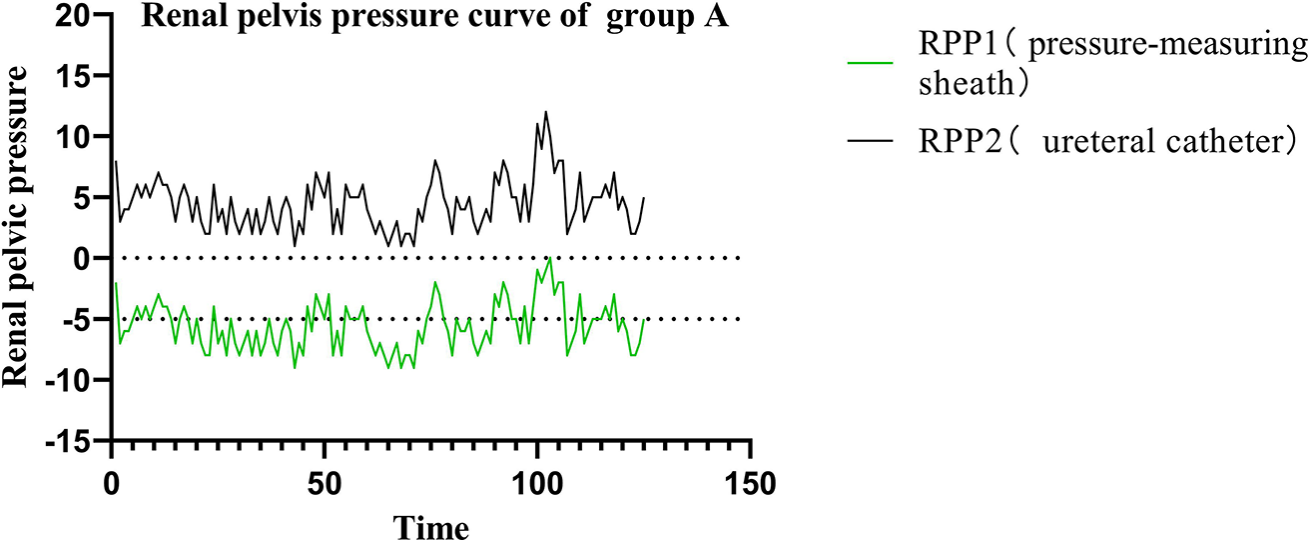
Curves of renal pelvic pressure in Group A

In Groups A and B, the RPP was measured by the pressure-measuring MPCNL suctioning sheath and the ureteral catheter. The preset infusion flow rate, intraluminal pressure control value, and intraluminal pressure warning value in Group A was 500 ml/min, -5 mmHg, and 20 mmHg, respectively. The RPP measured by the pressure-measuring MPCNL suctioning sheath was − 5.59 ± 1.95 mmHg, which was consistent with that measured by the ureteral catheter (4.46 ± 2.08 mmHg). The RPP fluctuated within − 5 mmHg from the preset intraluminal pressure (Fig. 3); that is, the RPP in Group A was always within a safe range (< 30 mmHg).

**Fig. 4:**
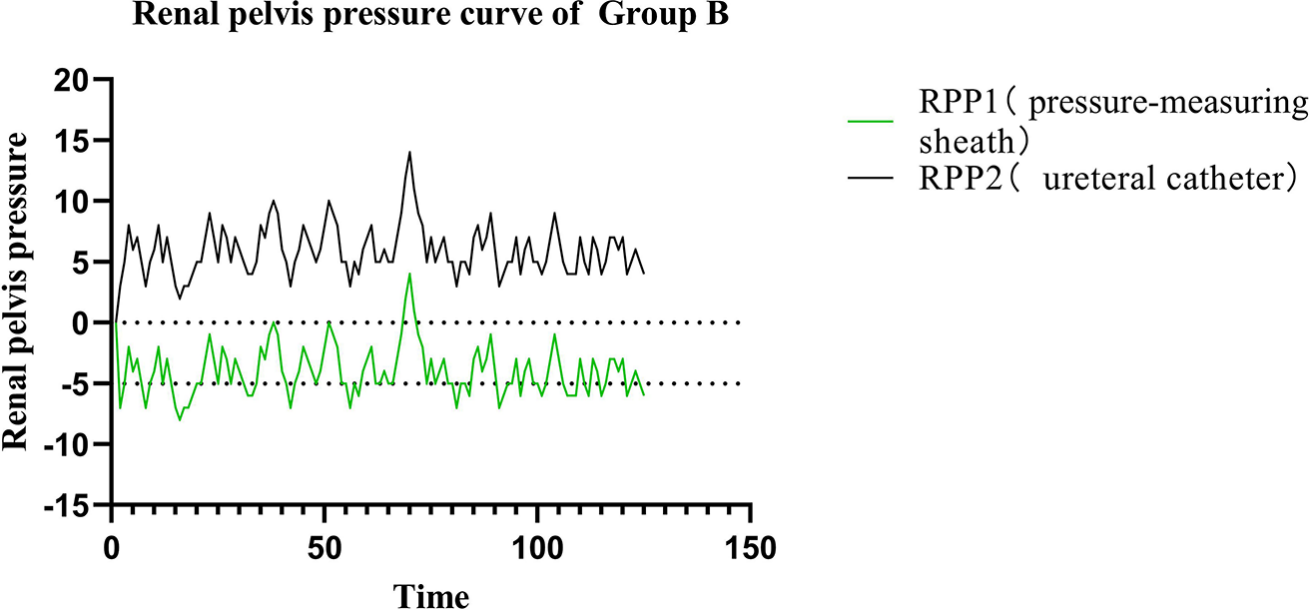
Curves of renal pelvic pressure in Group B

In Group B, the preset infusion flow rate, intraluminal pressure control value, and intraluminal pressure warning value was 750 ml/min, -5 mmHg, and 20 mmHg, respectively. The RPP measured by the pressure-measuring MPCNL suctioning sheath was − 4.00 ± 2.01 mmHg, which was consistent with the RPP measured by the ureteral catheter (5.92 ± 2.05 mmHg). The RPP fluctuated within − 5 mmHg from the preset intraluminal pressure (Fig. 4); thus, the RPP in Group B was always within a safe range.

**Fig. 5:**
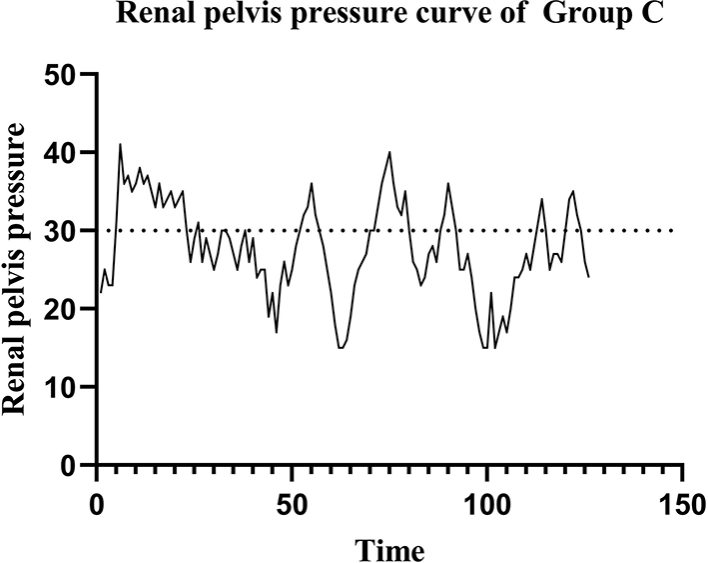
Curves of renal pelvic pressure in Group C

In Group C (the MPCNL group), the preset infusion flow rate was 500 ml/min, and the RPP measured by the ureteral catheter was 27.75 ± 5.98 mmHg, showing large fluctuations and exceeding the safe pressure of 30 mmHg approximately 30.9% of the time (Fig. 5).

**Fig. 6:**
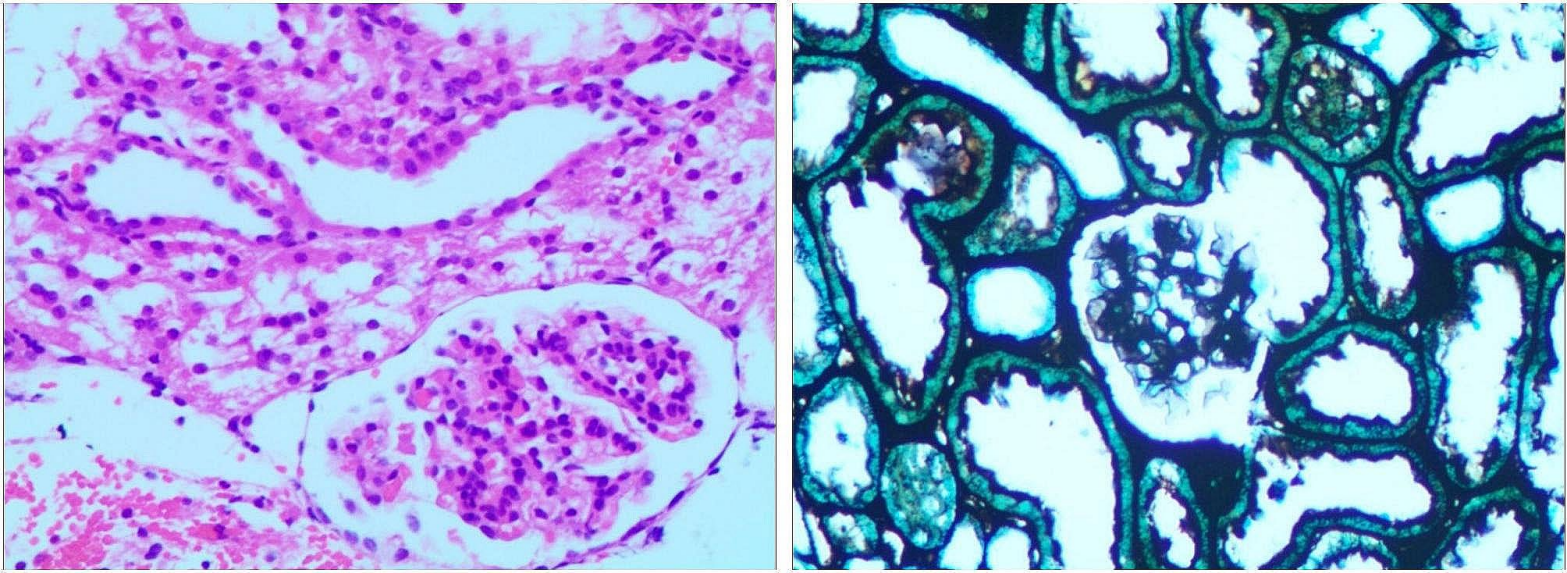
Postoperative renal histopathological HE staining (×200) and hexamine silver staining in Group C (× 200)

The intraoperative RPP monitored by the pressure-measuring MPCNL suctioning sheath in Group A and in Group B was maintained within the preset safe range (fluctuating within the preset intraluminal pressure control value) and was consistent with the RPP monitored by the ureteral catheter. The RPP in Group C fluctuated greatly and often exceeded the safe range. Postoperative renal histopathological examination revealed no evident structural damage to the nephrons in Group A or B. Postoperative color Doppler ultrasound showed no obvious perirenal extravasation in Group A or B. In contrast, postoperative histopathological examination of samples from Group C showed structural changes to the nephrons in 4/6 kidneys (Fig. 6), obvious protein exudate in the renal capsule, a thinned basement membrane of the renal capsule, and dilated renal tubules. Postoperative color Doppler ultrasound in Group C showed perirenal extravasation to different degrees. Although the same infusion flow rate was applied in Groups A and C, the RPP in Group C was significantly greater than that in Group A (*P* < 0.01) and fluctuated greatly.

## Discussion

In recent years, many new technological innovations and machines have been developed in the field of PCNL. The first innovation was the miniaturization of percutaneous renal channels. UMP features channels ranging from 11 to 13 Fr, while SMP, performed using equipment with an infusion negative-pressure suction system, has channels sized between 10 and 14 Fr. Additionally, a percutaneous needle nephroscope (Needle-Perc) with an outer sheath size of only 4.2 Fr has been developed and can serve as both a visual and puncture system. Another obvious innovation is the incorporation of negative-pressure suction in percutaneous renal sheaths, such as the percutaneous renal suction sheath invented by Song Leming, the ClearPetra sheath with negative-pressure aspiration capability, the Seplou percutaneous nephrostomy suction sheath, and the SMP sheath [10, 11]. The utilization of lithotripsy tools such as the Moses laser and thulium laser represents another facet of new technology. These innovations in PCNL also include the development of applications and training systems utilizing three-dimensional models, artificial intelligence and virtual reality and the implementation of robotic systems for PCNL [12].

The now widely used standard PCNL and MPCNL have both advantages and limitations. Standard PCNL combined with an ultrasonic ballistic lithotripsy (or double-catheter ultrasonic lithotripsy) system and negative-pressure suction can be used to remove stone fragments, which effectively reduces the RPP, the absorption of toxins and pyrogens during lithotripsy, extravasation, and the incidence of fever and sepsis. However, standard PCNL is traumatic and causes extensive blood loss, and it is difficult for the large devices used in standard PCNL to reach the narrow renal calyces. Therefore, there is a greater need to establish multiple tracts in standard PCNL [6, 13]. In standard PCNL, the RPP cannot be accurately regulated or monitored, and the risk of a high RPP cannot be eliminated. Compared with standard PCNL, MPCNL involves smaller tracts, less trauma, and less bleeding. However, in MPCNL, stones are mainly removed through high-pressure infusion, which is inefficient and easily causes a high RPP, in turn leading to intraoperative or postoperative fever, sepsis, septic shock, perirenal extravasation, and renal injury [14–17].

After years of research, based on the advantages of standard PCNL and MPCNL and seeking to improve upon their shortcomings, we manufactured a system integrating a pressure-measuring MPCNL suctioning sheath and an irrigation and suctioning platform with pressure monitoring and feedback control. This platform eliminates the limitations and preserves the advantages of standard PCNL and MPCNL, representing a new PCNL technique that combines MPCNL with intelligent RPP monitoring and control. This new PCNL technique is called IPC-MPCNL.

To verify the safety and reliability of the new system and the new technique, we simulated MPCNL in pigs. First, we tested the accuracy of the RPPs measured by the pressure-measuring MPCNL sheath by comparing them with those measured by the conventional ureteral catheter; if the two were consistent, then the RPPs measured by the pressure-measuring MPCNL sheath were accurate. Second, we tested whether the new technique and the new system could maintain the RPP within the preset safe range by measuring and recording the intraoperative RPP. The RPP, postoperative renal injury, and perirenal extravasation observed in IPC-MPCNL were compared with those in conventional MPCNL without suction or intelligent pressure control.

From the experimental results, the RPPs in IPC-MPCNL at different infusion flow rates (500 ml/min, 750 ml/min) fluctuated around the preset RPP value and were consistent with the RPPs measured by the ureteral catheter. At the same infusion flow rate (500 ml/min), the RPPs in conventional MPCNL fluctuated more intensively than did those in IPC-MPCNL and often exceeded the safe RPP limit, resulting in perirenal extravasation and renal injury.

In IPC-MPCNL, the RPP was maintained within the preset safe range under different infusion flow rates. The RPPs measured by the pressure-measuring MPCNL suctioning sheath were consistent with those measured by the ureteral catheter; that is, the RPPs measured by the two devices were consistent. Hence, our system, composed of a pressure-measuring MPCNL suctioning sheath and a medical irrigation and suctioning platform, is safe and reliable for monitoring and controlling the RPP. Since the RPP was maintained within a safe range during IPC-MPCNL, postoperative renal histopathology and renal color Doppler ultrasound showed no renal injury or perirenal extravasation.

Overall, the new PCNL technique based on the new system and method has the following characteristics: (1) The technique uses minitracts, and the MPCNL sheath integrates suctioning and pressure-measuring tracts. (2) In addition to the infusion function, the medical irrigation and suctioning platform has a pressure control system that integrates pressure monitoring, suctioning, and pressure feedback. Before the operation, the infusion flow rate, intraluminal pressure control value, and intraluminal pressure warning value needed for the operation can be set through the system so that the system can control the suction intensity and automatically and accurately maintain the RPP in a safe range through pressure feedback while maintaining the ideal infusion flow rate for the operation. Once the RPP exceeds the warning value for any reason, the system sounds an alarm or automatically shuts down to protect the patient, which simplifies the operation, thereby avoiding high RPP-induced complications such as urosepsis and extravasation. (3) This new system can ensure that the RPP is maintained within the preset safe range under different infusion flow rates. During stone fragmentation and removal, the infusion flow rate can be increased appropriately to increase the clarity of the surgical field for stone fragmentation, and suction can be used to increase the fluid circulation in the renal pelvis, allowing the stone fragments to be aspirated from the body along with the fluid. In this way, the efficiency of stone fragmentation and removal is greatly improved.

This study has several limitations. The experimental conditions were highly controlled in this study. Although our pressure measurement method is simple, convenient, and minimally invasive and does not require complicated steps or complex pressure-measuring devices, deviations in the measured pressures (by the hydraulic pressure measurement method) cannot be excluded because the long conduction distance from the pressure-measuring port to the pressure sensor may lead to the loss of pressure energy over distance and delay of the response. Furthermore, there may be risks of small stone fragments or blood clots blocking the pressure-measuring and suctioning tracts during stone fragmentation and removal in clinical practice. The movement of the pressure-measuring sheath during the operation will also lead to slight changes in pressure. Additionally, some postoperative parameters, such as hemoglobin loss, the presence of postoperative fever and sepsis, and inflammatory markers, were not evaluated. All these problems must be addressed. Since the working sheaths of UMP and SMP do not have pressure measurement channels, a similar study cannot be conducted with UMP or SMP .The safety and reliability of IPC-MPCNL and the system developed for IPC-MPCNL still need to be verified by large clinical trials.

## Conclusions

Animal experiments showed that IPC-MPCNL and the system developed for IPC-MPCNL are safe and effective and meet our design expectations. IPC-MPCNL can be applied to accurately monitor the RPP and maintain it within a preset safe range via suction.

## Data Availability

This study did not use public datasets. All data in the manuscript are available through the responsible corresponding author.

## Abbreviations

IPC-MPCNL: intelligent pressure-controlled minimally invasive percutaneous nephrolithotomy
MPCNL: minimally invasive percutaneous nephrolithotomy
RPP: renal pelvic pressure
PCNL: percutaneous nephrolithotomy
UMP: Ultra-mini-percutaneous nephrolithotomy
SMP: Super-Mini Percutaneous Nephrolithotomy
Needle-Perc: needle percutaneous nephroscopy
